# Oral health challenges during pregnancy in a semi-urban Indian population: A cross-sectional study in Valanchery, Kerala

**DOI:** 10.6026/973206300214464

**Published:** 2025-12-15

**Authors:** Shradha Anil Kuzhivilayil, Laxmy Rajmohan, Rahmath Beegam P.V

**Affiliations:** 1 General Dentist, Government District Hospital, Nilambur, Kerala, India; 2 Department of Obstetrics & Gynaecology, Government District Hospital, Nilambur, Kerala, India; 3 Department of Obstetrics & Gynaecology, CH Memorial Hospital, Valanchery, Malappuram, Kerala, India

**Keywords:** Pregnancy, oral health, gingivitis, dental caries, periodontitis, prenatal care, perinatal outcomes

## Abstract

Pregnancy is often characterized by an extreme physiological and hormonal state that makes them susceptible to oral diseases like gingivitis, caries and periodontitis. Therefore, it is of interest to review the rates and trend of oral health issues in 50 expectant mothers who were being attended to in CH Memorial Hospital, Valanchery, Kerala. Oral health status and behavior were measured using clinical examination and structured questionnaires. There was high prevalence of gingivitis (69%), dental caries (70%) and accumulation of plaque (65%) and low utilization of dental services by people (10%). Thus, we show that the application of oral health screening and education into the routine antenatal care is highly urgent to ensure the well-being of both the fetus and mother.

## Background:

Pregnancy is a temporary and dynamic physiological condition of significant endocrine, metabolic and immunological adaptations [1]. Although the presented adaptations are necessary to make the fetus develop successfully, they also make pregnant women more susceptible to numerous health problems, including those of oral cavities [2]. Increased hormone concentrations, especially estrogen and progesterone, change the microbiome of the mouth remarkably and modify the immune system of the host, predisposing pregnant women to inflammatory diseases such as pregnancy gingivitis and aggravating existing periodontal disease [3, 4]. The oral health issues of pregnancy have wide clinical ranges which include gingivitis, periodontitis, dental caries and dental erosions [5]. Pregnancy gingivitis, which is inflammation, erythema and bleeding of the gums is said to occur in 60-75 percent of pregnant women across the world, with the problem being highest in the second trimester [6]. Unattended, it may result in periodontitis, which is a chronic destructive inflammatory disorder of the tooth-supporting structures. Studies have also grown to enlighten an interesting relationship between maternal periodontitis and poor perinatal conditions, such as preterm birth, low birth weight and pre-eclampsia [7, 8]. The hypothetical mechanism is that systemic oral pathogen dissemination (e.g., Porphyromonas gingivalis) and inflammatory mediators dispersal (e.g., TNF-a, IL-6) can initiate pathological processes at the fetoplacental unit [9, 10]. Dental caries is another prevalent concern, with studies indicating a higher incidence among pregnant women compared to their non-pregnant counterparts [11]. Contributing factors include changes in dietary habits (e.g., increased carbohydrate consumption), reduced salivary pH and acid erosion from nausea-induced vomiting (hyperemesis gravidarum) [12]. Maternal caries not only impacts the mother's quality of life but also poses a risk for vertical transmission of cariogenic bacteria, such as Streptococcus mutans, to the infant, thereby increasing the risk of early childhood caries [13]. Despite the established links between oral health and overall maternal-fetal well-being, a significant gap persists in the integration of dental care into routine antenatal protocols, particularly in low- and middle-income settings [14]. Many pregnant women do not receive oral health screening or counseling and utilization of professional dental services remains low due to barriers such as lack of awareness, financial constraints and misconceptions regarding the safety of dental procedures during pregnancy [15]. In India, regional data on the oral health status of pregnant women are limited, especially from semi-urban and rural areas where access to specialized healthcare may be constrained. Therefore, it is of interest to assess the prevalence of common oral health conditions-namely gingivitis, dental caries and plaque accumulation-among pregnant women attending an outpatient clinic in Valanchery.

## Materials and Methods:

## Study design and setting:

A cross-sectional observational study was conducted over a period of three months, from Nov 2024 to Dec 2024. The study was carried out at the Antenatal Outpatient Department (OPD) of CH Memorial Hospital, a secondary care facility in Valanchery, Malappuram district, Kerala, India.

## Study population and sampling:

The study population consisted of pregnant women attending the antenatal OPD for routine check-ups. A sample size of 50 participants was determined using a convenience sampling method.

[1] Inclusion Criteria: All consenting pregnant women, irrespective of their gestational age, parity, or pre-existing medical conditions, were included in the study.

[2] Exclusion Criteria: Women who were unwilling to provide informed written consent, those with severe systemic illnesses that precluded a routine oral examination or those experiencing acute dental emergencies requiring immediate intervention were excluded.

## Data collection procedure:

After obtaining informed written consent, each participant was administered a pre-tested, structured questionnaire by a trained investigator to collect data on sociodemographic characteristics (age, education, occupation), obstetric history (trimester, parity) and oral health-related behaviours (frequency of tooth brushing, use of interdental aids, history of dental visits). Following the questionnaire, a comprehensive intraoral examination was performed by a single calibrated examiner (a dental house surgeon) under standardized conditions using a plane mouth mirror and a Community Periodontal Index (CPI) probe under adequate illumination. The examination was conducted with the participant seated comfortably in a dental chair.

## Clinical assessment tools:

Oral health status was determined using the following standardized indices:

## Gingivitis:

The severity of gingivitis was determined by using the Loe and Silness Gingival Index (GI). The scores are 0 (normal gums) to 3 (massive inflammation, with spontaneous bleeding). In the current research, a respondent would have been diagnosed with gingivitis when the mean GI score was greater than 1.0.

## Plaque accumulation:

The Silness and Loe Plaque Index (PI) was applied to rate the amount of soft debris on the teeth. The scoring goes between 0 (no plaque) to 3 (abundance of plaque within the gingival pocket). The mean PI score <1.0 was taken as a sign of major accumulation of plaque.

## Dental caries:

Dental Caries experience was measured using the Dental Caries, The Decayed, Missing and Filled Teeth (DMFT) index according to World health Organization (WHO) criteria. Each person was given a total DMFT score which was the summation of the decayed (D), missing because of caries (M) and filled teeth (F).

The people diagnosed to have serious oral health problems were given oral hygiene education and sent out to the dental department to be evaluated and handled further.

## Statistical analysis:

The data obtained were typed and analyzed in a spreadsheet in Microsoft Excel and in the Statistical Package of the Social Sciences (SPSS) version 25.0 (IBM Corp., Armonk, NY). Data was summarized with the help of descriptive statistics; continuous variables (e.g., age, DMFT score) were summarized to mean +- standard deviation (SD), whereas categorical variables (e.g., prevalence of gingivitis, education level) were summarized with frequencies and percentages. The Chi-square test was employed to determine the relationship between categorical variables, i.e., oral hygiene practices and the existence of clinical conditions (plaque, gingivitis). A p-value of less than 0.05 was taken to be of significant value.

## Results:

A total of 50 pregnant women participated in the study. The mean age of the participants was 26.4 ± 4.2 years, with ages ranging from 20 to 35 years. The majority of women were in their second (44%) or third (38%) trimester of pregnancy. Over half of the participants (54%) had completed secondary education and most were homemakers (72%). Detailed sociodemographic and clinical characteristics are presented in Table 1 (see PDF). The prevalence of oral health morbidities was notably high among the study participants. Gingivitis was diagnosed in 34 women (69%), while dental caries was present in 35 women (70%). Significant plaque accumulation (PI score ≥1.0) was observed in 32 women (65%). The mean DMFT score for the cohort was 4.8 ± 2.1. Regarding oral hygiene behaviors, while most women (78%) reported brushing their teeth at least once a day, only 30% brushed twice daily. The use of interdental cleaning aids like floss was virtually non-existent (2%). A critical finding was that 80% (n=40) of the participants required further dental assessment and treatment, yet only 10% (n=50) had consulted a dentist during their pregnancy. These findings are summarized in Table 2 (see PDF). A Chi-square analysis was performed to determine the association between self-reported brushing frequency and the presence of plaque and gingivitis. A statistically significant association was found between brushing twice daily and lower prevalence of both plaque accumulation and gingivitis. Participants who brushed their teeth only once a day or less were significantly more likely to have notable plaque buildup (p=0.021) and clinical gingivitis (p=0.035) compared to those who brushed twice a day. These associations are detailed in Table 3 (see PDF).

## Discussion:

This cross-sectional study provides critical insights into the oral health status of pregnant women in a semi-urban setting in Kerala, revealing a high prevalence of preventable oral diseases and a significant deficit in professional dental care utilization. The findings underscore the urgent need for targeted public health interventions to bridge this gap. The observed prevalence of gingivitis in our cohort was 69%, a figure that aligns with the global range of 60-75% reported in various epidemiological studies and systematic reviews [6, 16]. This high incidence is primarily attributed to the physiological surge in progesterone and estrogen during pregnancy, which increases gingival vascular permeability and exaggerates the inflammatory response to dental plaque [17]. The significant association found in our study between suboptimal brushing frequency and higher gingivitis rates (p=0.035) reinforces the concept that while hormonal changes create a permissive environment, poor oral hygiene remains the primary etiological factor [4]. Without proper intervention, this condition can progress to periodontitis, which has been robustly linked to adverse pregnancy outcomes. A meta-analysis by Khader and Ta'ani demonstrated that maternal periodontitis significantly increases the risk of preterm birth and low birth weight [8]. Similarly, the prevalence of dental caries (70%) in our study population is alarming and consistent with findings from other regions in India and globally, where pregnancy is identified as a high-risk period for caries development [11, 18]. The mean DMFT score of 4.8 indicates a moderate but cumulative burden of dental disease. The underlying reasons are multifactorial, including increased frequency of snacking on carbohydrates, acidic challenges from morning sickness and potential changes in salivary composition and flow rate [12, 19]. The presence of untreated carious lesions in a majority of affected women is particularly concerning, as it not only compromises maternal health but also establishes a reservoir for cariogenic bacteria that can be vertically transmitted to the infant, predisposing them to early childhood caries [13]. Perhaps the most striking finding of this study is the profound disparity between the need for dental care and its actual utilization. While 80% of participants required further dental assessment or treatment, a mere 10% had sought any form of professional dental help during their pregnancy. This low rate of dental visits is a pervasive issue reported worldwide. Studies from the United States, for instance, have shown that only 22-34% of pregnant women consult a dentist [15]. The barriers in our local context are likely multifaceted, stemming from a lack of awareness among both patients and non-dental healthcare providers, financial constraints, fear of dental procedures and persistent myths about the safety of dental treatment during pregnancy [20]. This highlights a critical failure in the healthcare system to integrate oral health into the standard prenatal care paradigm. The American College of Obstetricians and Gynecologists (ACOG) explicitly recommends routine oral health assessment and necessary dental treatment at any stage of pregnancy, emphasizing its safety and importance [21]. Our study demonstrated a clear, statistically significant link between better oral hygiene practices (brushing twice daily) and improved clinical outcomes (lower plaque and gingivitis). This simple, evidence-based finding provides a powerful foundation for preventive counseling. Educating expectant mothers on the importance of meticulous oral hygiene, including effective brushing techniques and the benefits of interdental cleaning, can substantially mitigate the hormone-mediated oral changes of pregnancy [22]. This study has some limitations. The cross-sectional design prevents the establishment of causality between risk factors and outcomes. The small sample size and convenience sampling method from a single center may limit the generalizability of our findings to the broader population of pregnant women in Kerala. Furthermore, data on oral hygiene practices were self-reported and may be subject to social desirability bias. Future research should employ longitudinal designs with larger, more diverse cohorts to track changes in oral health across trimesters and evaluate the efficacy of structured interventional programs.

## Conclusion:

Maintaining optimal oral health during pregnancy is vital for the well-being of both mother and child. Early detection, preventive care and health education should be integral components of antenatal programs. Thus, strengthening collaboration between dental and obstetric healthcare providers can significantly reduce the burden of oral diseases and improve overall pregnancy outcomes.
